# Rosai-Dorfman disease disguised as BRAF V600E-positive histiocytosis – a diagnostic pitfall

**DOI:** 10.1007/s44313-025-00061-x

**Published:** 2025-02-28

**Authors:** Aishwarya Ravindran, Gaurav Goyal, Diana Morlote, Alexander C. Mackinnon

**Affiliations:** 1 Division of Laboratory Medicine – Hematopathology, Department of Pathology|West Pavilion 230-D, The University of Alabama at Birmingham, 619 19th St. S., Birmingham, AL 35249-7331 USA; 2 Division of Hematology and Medical Oncology, University of Alabama at Birmingham, Alabama, USA; 3 Division of Genomic Diagnostics & Bioinformatics, University of Alabama at Birmingham, Alabama, USA

**Keywords:** Rosai-Dorfman disease, BRAF, Next-generation sequencing

A 45-year-old female presented with multifocal marrow-replacing lesions, which were identified incidentally on magnetic resonance imaging of the cervical spine during evaluation of a minor neck injury. Serum monoclonal protein studies and bone marrow biopsies were negative for a plasma cell neoplasm. Further radiologic evaluation revealed retroperitoneal and inguinal lymphadenopathy, the biopsy of which revealed nodal parenchymal effacement by sheets of atypical histiocytes with enlarged round/oval nuclei, conspicuous nucleoli, pale chromatin, and abundant pale eosinophilic cytoplasm with emperipolesis (Fig. 1A, Hematoxylin and eosin stain, inset: red arrow indicates lesional histiocyte nuclei; black arrow indicates lymphocytes in the cytoplasm of the lesional histiocytes). These lesional histiocytes were positive for CD163 (Fig. 1B, × 200), S100 (Fig. 1C, × 200), cyclin D1 (Fig. 1D, × 200, red arrows show strong nuclear and weak cytoplasmic expression), and OCT2 (Fig. 1E, × 200, red arrow indicates lesional histiocytes; black arrow indicates background B-cells/plasma cells) and negative for CD1a and langerin, supporting a diagnosis of Rosai-Dorfman disease (RDD). Immunostaining for BRAF V600E revealed weak cytoplasmic granular positivity (Fig. 1F). Next-generation sequencing (NGS) (Oncomine Precision Assay; Thermo Fisher Scientific, Waltham, MA, USA) revealed no pathogenic variants, including in *BRAF *gene. Given the discordant immunohistochemistry and NGS results, the sequencing data was manually examined, which confirmed the absence of low-abundance reads containing the c.1799T > A missense variant encoding V600E. False-positive BRAF V600E immunostaining is extremely rare, and the lack of strong cytoplasmic expression, as in this case, should raise suspicion of non-specific immunoreactivity in lesional histiocytes, necessitating confirmatory molecular analysis.

**Fig. 1:**
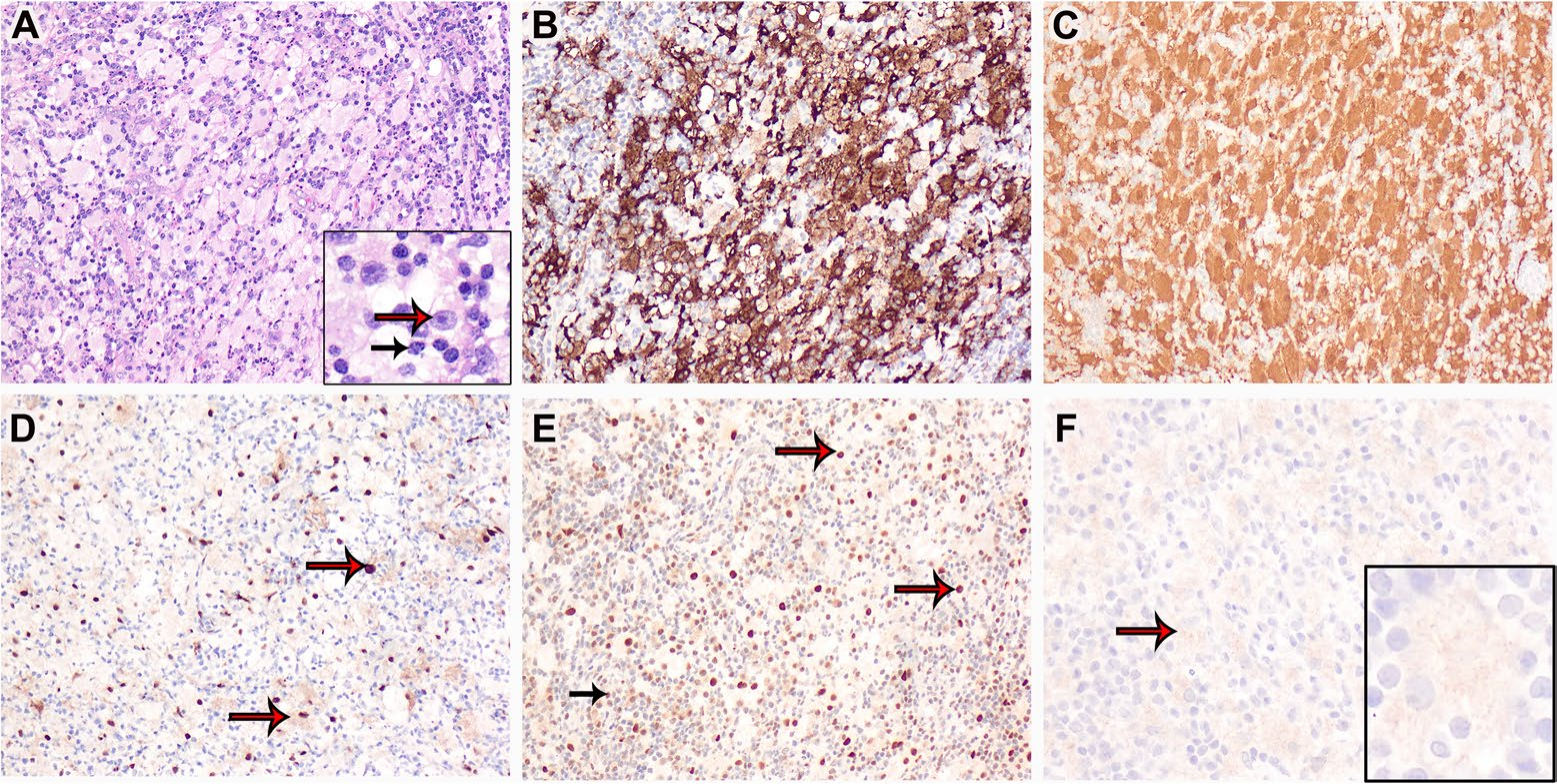
.

Approximately 30–50% of RDD cases harbor MAPK pathway mutations; therefore, RDD is classified as a distinct histiocytic neoplasm per the 2022 World Health Organization (5th edition) and the International Consensus Classification of hematopoietic tumors. *BRAF* mutations, although extremely rare in RDD, have been reported in codons 600 (V600E), 472 (Y472C), and 188 (R188G). While RDD was historically considered a reactive and self-limiting histiocytosis, given the contemporary data and the availability of Food and Drug Administration approved therapies for adult histiocytic neoplasms, it is recommended to perform NGS to identify symptomatic patients who may be suitable for targeted therapy.

## Data Availability

No datasets were generated or analysed during the current study.

